# Fe–Doped TiO_2_–Carbonized Medium–Density Fiberboard for Photodegradation of Methylene Blue under Visible Light

**DOI:** 10.3390/ma14174888

**Published:** 2021-08-27

**Authors:** Justin Alfred Pe, Sung-Phil Mun, Min Lee

**Affiliations:** 1 Department of Wood Science and Technology, Jeonbuk National University, Jeonju 54896, Korea; justinpe@jbnu.ac.kr; 2 Department of Wood Products, National Institute of Forest Science, Seoul 02455, Korea; mlee81@korea.kr

**Keywords:** medium-density fiberboard, Fe–doped TiO_2_, photodegradation, methylene blue, visible light

## Abstract

Fe–doped titanium dioxide–carbonized medium–density fiberboard (Fe/TiO_2_–cMDF) was evaluated for the photodegradation of methylene blue (MB) under a Blue (450 nm) light emitting diode (LED) module (6 W) and commercial LED (450 nm + 570 nm) bulbs (8 W, 12 W). Adsorption under daylight/dark conditions (three cycles each) and photodegradation (five cycles) were separately conducted. Photodegradation under Blue LED followed pseudo-second-order kinetics while photodegradation under commercial LED bulbs followed pseudo-first-order kinetics. Photodegradation rate constants were corrected by subtracting the adsorption rate constant except on the Blue LED experiment due to their difference in kinetics. For 8 W LED, the rate constants remained consistent at ~11.0 × 10^−3^/h. For 12 W LED, the rate constant for the first cycle was found to have the fastest photodegradation performance at 41.4 × 10^−3^/h. After the first cycle, the rate constants for the second to fifth cycle remained consistent at ~28.5 × 10^−3^/h. The energy supplied by Blue LED or commercial LEDs was sufficient for the bandgap energy requirement of Fe/TiO_2_–cMDF at 2.60 eV. Consequently, Fe/TiO_2_–cMDF was considered as a potential wood-based composite for the continuous treatment of dye wastewater under visible light.

## 1. Introduction

Medium-density fiberboard (MDF), one of the wood-based panels with various physical properties and grades, is an extremely versatile building product that is commonly used in many home and professional projects including furniture, flooring, decorative projects, doors, and door frames. MDFs can have additional enhanced properties by their carbonization at high temperatures (600–1400 °C). Carbonized medium-density fiberboards (cMDFs) were first prepared by Kercher and Nagle for electrical applications [1]. However, the cMDF prepared was for laboratory applications, not for industrial utilization. Thus, a large-sized, crack- and twist-free cMDF was developed using a pressing carbonization method [2]. The cMDFs exhibited outstanding removal properties for volatile organic compounds (VOCs) such as formaldehyde and ammonia; however, the cMDFs showed poor removal properties for aromatic VOCs such as benzene, toluene, and xylene [3]. Mun and Park prepared TiO_2_–cMDF intended to improve its aromatic VOCs removal performance [4]. TiO_2_ has proved to be an effective material for the degradation of VOCs due to its strong oxidation properties, high stability, and cost effectiveness [5]. Lee et al. also prepared two different types of TiO_2_–cMDFs for the removal of aromatic VOCs, especially toluene: (1) one-step preparation of TiO_2_–cMDF through in situ formation from a titanium tetraisopropoxide precursor on the surface of MDF via carbonization [6] and (2) synthesis of TiO_2_ by sol-gel method and then application to the surface of cMDF [7]. These TiO_2_–cMDFs exhibited outstanding removal performance for formaldehyde, one of the major VOCs that cause sick house syndrome, and toluene, a hardly degradable aromatic VOC, through adsorption and photodegradation under UV irradiation [6,7]. However, the efficiency of the TiO_2_/UV processes are limited by their fast recombination of electron-hole pairs and wide band gap, which restricts light absorption to the ultraviolet region [8]. Metal doping inhibits the recombination of electron-hole pairs and extends light absorption to the visible region [9]. Moreover, doping TiO_2_ photocatalysts with metals such as Fe enhances their photocatalytic activity against pollutants [10,11,12]. The visible light responsive TiO_2_–cMDFs were also prepared by Lee et al. through metal (Fe or Co) doping and investigated their photodegradation performance under visible light [13]. The Fe or Co/TiO_2_–cMDF removed 100% of toluene gas under a commercial fluorescent lamp. No difference was observed between the toluene degradation performances of Fe and Co/TiO_2_–cMDF.

Previously, the authors evaluated the photocatalytic activity of TiO_2_–cMDF against aqueous methylene blue (MB) dye solution under different sources of UV light (UV-C lamp and UV LEDs) [14,15]. The results showed that TiO_2_–cMDF under a UV-C lamp and UV-A LED practically removed MB even after repeated use. However, no studies regarding the photocatalytic activity of the newly developed Fe/TiO_2_–cMDF in aqueous solution were reported. Hence, the purpose of this study was to investigate the adsorption and photodegradation of MB dye with Fe/TiO_2_–cMDF under Blue LED (450 nm) and commercial LED (450 nm + 570 nm) bulbs (8 W, 12 W). In addition, the authors also investigated the reusability of the Fe/TiO_2_–cMDF for future applications in continuous wastewater treatment systems.

## 2. Materials and Methods

### 2.1. Materials

Titanium tetraisopropoxide (Ti-tip, 98%) and isopropyl alcohol (IPA, 99.5%) were purchased from Daejung Chemicals (Siheung, Korea). Iron (III) nitrate nonahydrate (98%) was purchased from Junsei Chemical (Tokyo, Japan). The Blue LED modules were provided by the Korea Photonics Technology Institute (Gwangju, Korea). The commercial LED bulbs (Dongyang Tospo Lighting Optoelectronic, Dongyang, China) were purchased locally.

### 2.2. Preparation and X-ray Diffraction of Fe-Doped TiO_2_

The preparation of Fe–doped TiO_2_ was reported earlier [13]. A photocatalyst precursor Ti-tip was dissolved in 50% *v/v* IPA (Solution A). A 1 mol Fe dopant was dissolved in IPA (Solution B) and transferred to a separatory funnel. A calculated volume of Solution B was added in a dropwise manner to Solution A to achieve a 0.1 mol% Fe/Ti-tip mixture.

The mixture was thoroughly mixed by stirring and sonication. To characterize the Fe-doped TiO_2_, the solvent was removed by a stream of nitrogen. The dry Fe-doped TiO_2_ was pulverized and calcined at 800 °C for 2 h. The TiO_2_ crystalline structure of Fe-doped TiO_2_ was determined by X-ray powder diffraction (XRD, X’Pert powder, Malvern Pananalytical, Malvern, UK) under the conditions of 40 kV and 30 mA in the range of 15° to 80° from the starting angle.

### 2.3. Preparation of Fe/TiO_2_–cMDF

Approximately 7 g of the Fe/Ti-tip mixture was applied on the surface of MDF specimens of dimensions 260 mm (L) × 100 mm (W) × 7 mm (T) via brush coating method. The Fe/Ti-tip-treated MDFs were air-dried in a fume hood and further dried in a convection oven at 60 °C for 3 h.

**Figure 1:**
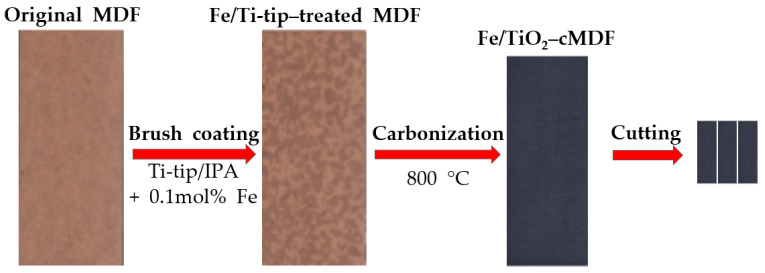
Preparation of the Fe/TiO_2_–cMDF panel and slabs.

The dry Fe/Ti-tip-treated MDFs were carbonized in an electric furnace with a ramping rate of 100 °C/h to 800 °C and held for 2 h. The preparation of Fe/TiO_2_–cMDF is shown in Figure 1.

### 2.4. UV/Vis-Diffuse Reflectance Spectroscopy of Fe/TiO_2_–cMDF

The ultraviolet-visible absorption spectra of Fe/TiO_2_–cMDF were measured by a UV/Vis-Diffuse reflectance spectrophotometer (UV/Vis-DRS, S-4100, Scinco, Seoul, Korea). The optical bandgap energy (E_g_) was estimated using the Kubelka–Munk function by plotting the [F(R)hν]^n^ vs. energy, where R is reflectance, F(R) is the Kubelka–Munk function, h is Planck’s constant (J∙s), ν is frequency (1/s), and n denotes the nature of semiconductor (n = 2, since rutile-type TiO_2_ is a direct bandgap semiconductor).

### 2.5. Evaluation for the Adsorption of MB Using Fe/TiO_2_–cMDF

**Figure 2:**
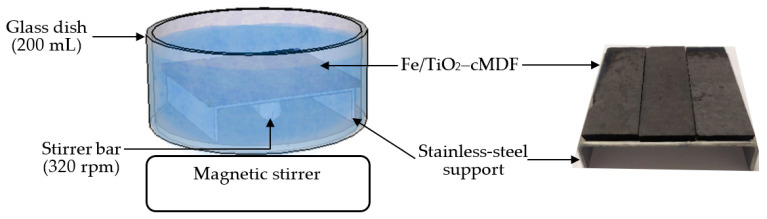
Representation of the adsorption experiment.

The resulting Fe/TiO_2_–cMDF panel prepared from the method above was cut into slabs of dimensions 60 mm (L) × 20 mm (W) × 3 mm (T). Three Fe/TiO_2_–cMDF slabs were fixed on a stainless-steel support with a polyethylene hot-melt adhesive. The slabs were then immersed in 175 mL of 10 ppm MB (Yakuri Pure Chemicals, Kyoto, Japan) solution in a 200 mL glass dish under magnetic stirring (Figure 2). Adsorption experiments were performed in daylight and dark conditions with three cycles each. After each cycle, the solution was discarded and a fresh batch of 175 mL of 10 ppm MB solution was added.

### 2.6. Evaluation for the Photodegradation of MB

**Figure 3:**
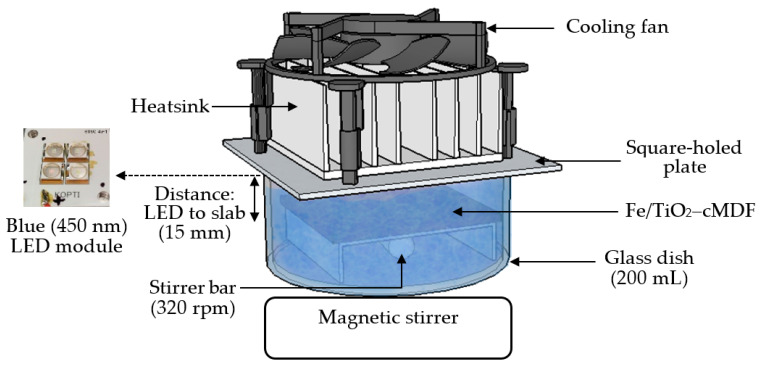
Representation of the photodegradation experiment under Blue LED.

For the photodegradation experiment under Blue LED (450 nm, 6 W), three new Fe/TiO_2_–cMDF slabs were immersed in 175 mL of 10 ppm MB solution in a 200 mL glass dish under magnetic stirring at a temperature range of 24–25 °C. One LED module was attached to an aluminum heatsink with a cooling fan and placed above a square-holed plate (Figure 3). The LED modules were connected to a DC regulated power supply. The Blue LED operated at 7.6 V and 200 mA. The photodegradation of MB was performed for five cycles in dark conditions only. After each cycle, the solution was discarded and a same amount of fresh MB solution was used each time.

**Figure 4:**
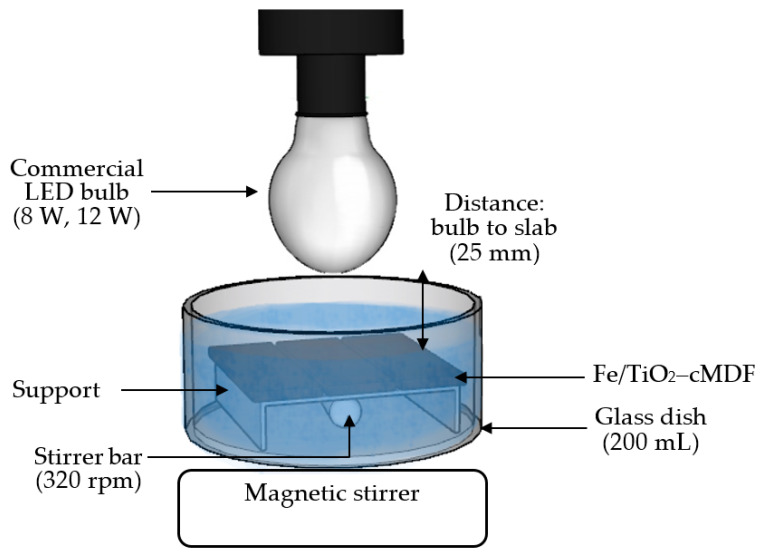
Representation of the photodegradation experiment under commercial LED bulbs.

For the photodegradation experiment under commercial LED (450 nm + 570 nm, 80 lm/W, 6500 K) bulbs (640 lm/8 W, 960 lm/12 W), the bulb was fixed on a makeshift support and placed above the glass dish (Figure 4). The photodegradation of MB was similarly performed to the Blue LED experiment.

The removal of MB was determined by the decrease in absorbance at the maximum wavelength (665 nm) using a visible spectrophotometer (Optizen 3220, Mecasys, Daejeon, Korea). The MB removal was calculated using Equation (1):

MB removal (%) = [(C_o_ − C_t_)/C_o_] × 100 (1)

where C_o_ is the initial concentration (ppm) and C_t_ denotes the concentration (ppm) at time t (h).

The nonlinear plots of the MB removal were transformed into linear plots using normalization through exponential (Equation (2)) function for pseudo-first-order kinetics or hyperbolic (Equation (3)) function for pseudo-second-order kinetics. The rate constants were derived from the slopes of the following Equations (2) and (3):

ln C_t_ = ln C_o_ − *k*_1_t (2)

(1/C_t_) = (1/C_o_) + *k*_2_t (3)

where *k*_1_ is pseudo-first-order rate constant (1/h) and *k*_2_ is pseudo-second-order rate constant (1/ppm∙h).

### 2.7. Elemental Distribution on the Surface of Fe/TiO_2_–cMDF

The surface of Fe/TiO_2_–cMDF was examined using scanning electron microscopy–energy dispersive spectrometer (SEM–EDS) (Supra 40VP, Carl Zeiss AG, Oberkochen, Germany) at the Center for University-wide Research Facility, Jeonbuk National University. The surface images were taken at 350× magnification and a total of 18 data points (three slabs, three equidistant spots per slab, two different SEM scans per spot) were collected for analysis.

## 3. Results and Discussion

### 3.1. Crystallinity of Fe-Doped TiO_2_

**Figure 5:**
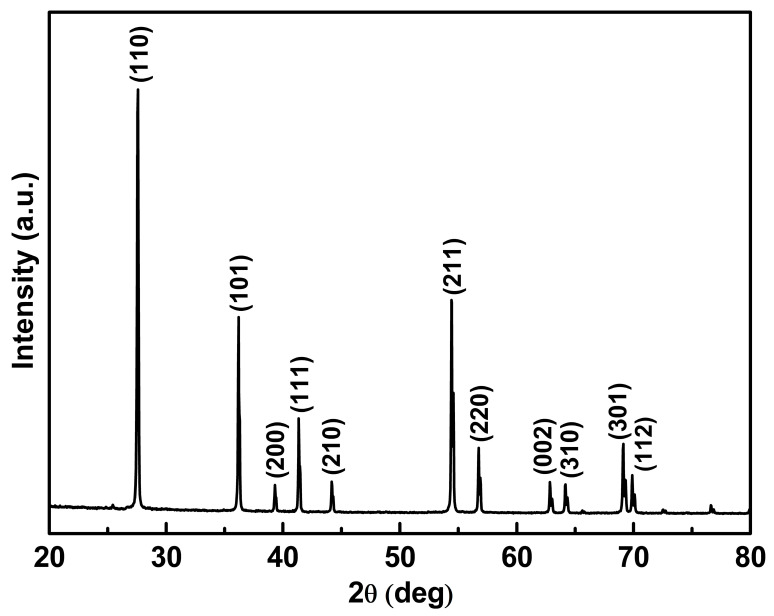
XRD spectrum of Fe-doped TiO_2_.

Figure 5 shows the XRD spectrum of a 0.1 mol% Fe-doped TiO_2_ photocatalyst calcined at 800 °C for 2 h. The XRD peaks showed that rutile-type TiO_2_ formed during the carbonization at 800 °C. The XRD peaks for Fe-doped TiO_2_ is in agreement with the characteristic peaks of rutile-type TiO_2_ at 2θ values of 27.6°, 36.3°, 39.2°, 41.3°, 44.0°, 54.4°, 56.6°, 62.9°, 64.0°, 69.0°, and 69.8° (JCPDS Card no. 21-1276). In addition, the XRD spectrum did not detect the presence of Fe, probably because of the low amount of Fe inclusions in the TiO_2_ lattice.

**Figure 6:**
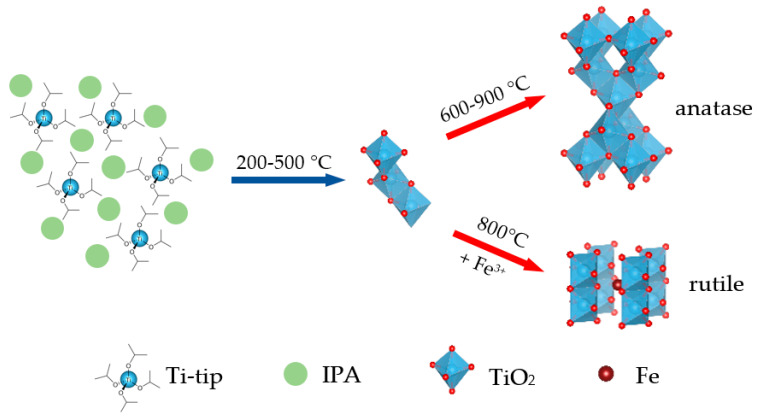
Graphical representation for the formation of anatase TiO_2_ and rutile TiO_2_ doped with Fe.

Anatase was the favored crystalline structure of the Ti-tip-treated cMDF carbonized at 600–900 °C; however, carbonization beyond 900 °C preferred the formation of rutile [6]. In the case of Fe-doped TiO_2_, Fe may have prevented the formation of anatase by inclusion of Fe in the TiO_2_ lattice and favored the formation of rutile (Figure 6).

### 3.2. Optical Bandgap Energy of Fe/TiO_2_–cMDF

**Figure 7:**
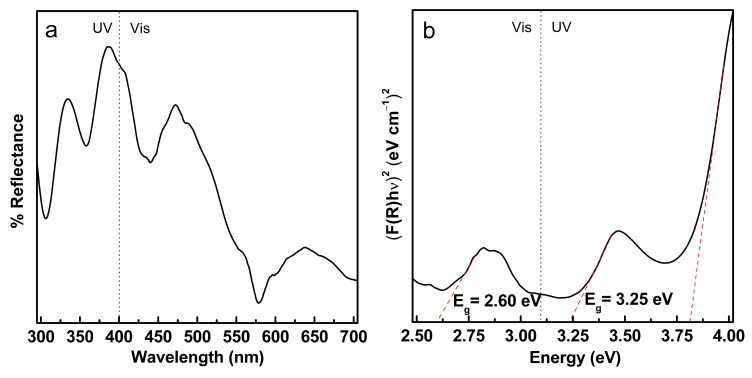
UV/Vis-DRS of Fe/TiO_2_–cMDF (**a**) Diffuse reflectance spectrum (**b**) Kubelka–Munk plot.

Figure 7a shows the UV/Vis-DRS spectrum in reflectance mode and Figure 7b shows the transformed plot using the Kubelka–Munk equation. The optical bandgap energies (E_g_) for rutile-type TiO_2_ and anatase-type TiO_2_ are 3.01 and 3.20 eV, respectively [8,16]. The E_g_ for Fe/TiO_2_–cMDF in the visible region was found to be 2.60 eV, lower than the E_g_ for rutile-type TiO_2_. Moreover, further E_g_s found in the Kubelka–Munk plot appeared in the UV region at 3.25 eV.

### 3.3. Adsorption Characteristics of MB on Fe/TiO_2_–cMDF

**Figure 8:**
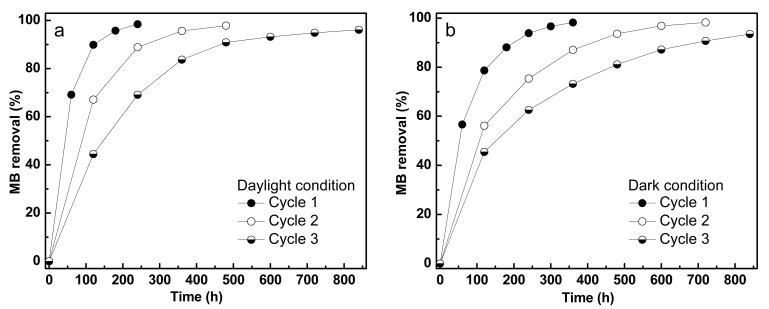
Adsorption kinetics of MB removal in (**a**) daylight and (**b**) dark conditions.

Various carbonized wood and wood-based materials together with TiO_2_–cMDFs are known to have high BET surface areas, ranging from 100 to 850 m^2^/g [4,17]. Considering the adsorption capabilities of carbonized wood-based materials, including TiO_2_–cMDFs, Fe/TiO_2_–cMDF was subjected to three full adsorption cycles with MB solution in daylight and dark conditions. The adsorption in daylight condition was almost completed in 258 h while adsorption in dark condition took 396 h. The second and third adsorption cycles for both daylight and dark conditions took longer times. The adsorption in daylight conditions (Figure 8a) followed pseudo-first-order kinetics and the rate constants decreased from 12.9 × 10^−3^/h (first cycle) to 3.0 × 10^−3^/h (third cycle). The adsorption in dark conditions (Figure 8b) also followed pseudo-first-order kinetics and the rate constant decreased from 8.5 × 10^−3^/h (first cycle) to 2.6 × 10^−3^/h (third cycle). The results showed that daylight conditions were faster than dark conditions by a difference of 4.4 × 10^−3^/h from their initial cycles (Table 1). Based on these results, the Fe/TiO_2_–cMDF was confirmed to be responsive to ambient light from the laboratory. Thus, the photodegradation experiments were performed in dark conditions only.

### 3.4. Photodegradation Performance of Fe/TiO_2_–cMDF

#### 3.4.1. Photodegradation Performance of Fe/TiO_2_–cMDF under Blue LED

**Figure 9:**
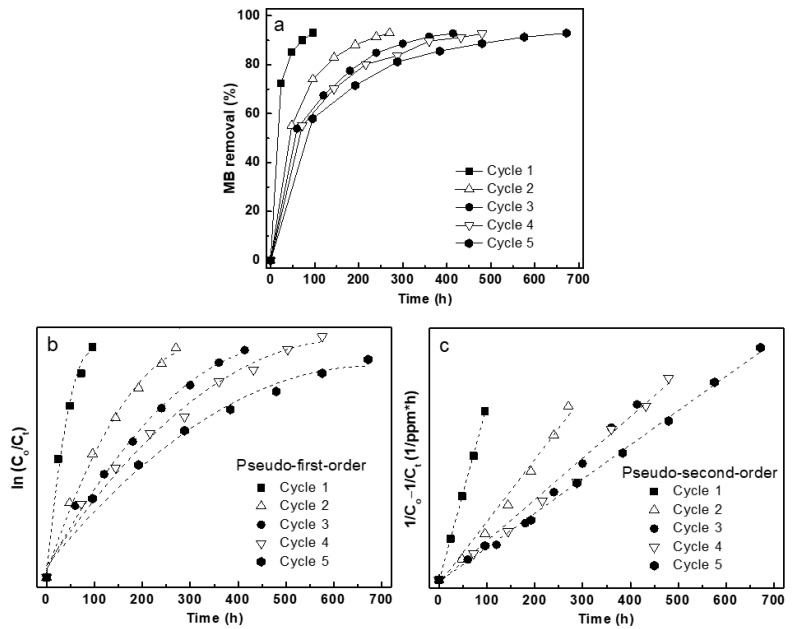
Photodegradation kinetics of MB under Blue LED: (**a**) MB removal plot, (**b**) normalized plot for pseudo-first-order, and (**c**) normalized plot for pseudo-second-order.

The photodegradation experiments were performed without pre-adsorption using a fresh set of Fe/TiO_2_–cMDF slabs for five cycles. The photodegradation cycle under a Blue LED module was deemed completed when MB removal reached 93% (Figure 9a). Normalization of the MB removal plot either follows the pseudo-first-order kinetics with a slope of ln (C_o_/C_t_) (Figure 9b) or pseudo-second-order kinetics with a slope of 1/C_o_ − 1/C_t_ (Figure 9c). The plot that assumes a linear trend is considered the adsorption kinetic model. In this case, photodegradation of MB under Blue LED followed pseudo-second-order kinetics.

The photodegradation kinetics of MB removal under Blue LED is listed in Table 2. The rate constant for the first cycle was found to be 10.7 × 10^−3^/ppm∙h. Since this experiment was a direct photodegradation without pre-adsorption, the fast rate constant for the first cycle was presumably caused by simultaneous adsorption and photodegradation. A decrease in the rate constant for the second cycle was also observed; the difference was smaller than that of the first cycle. The rate constants for the third to fifth cycle were very similar, ranging from 2.6 × 10^−3^/ppm∙h to 2.1 × 10^−3^/ppm∙h. This indicates that the adsorption of MB was almost completed as the numbers of photodegradation cycles were increased. The rate constants for the actual photodegradation could be calculated by obtaining the difference between the photodegradation rate constants and the adsorption rate constants in dark conditions. However, obtaining the difference between the photodegradation rate constants under Blue LED (pseudo-second-order) and the adsorption rate constants (pseudo-first-order) are inappropriate since they are based on different kinetic models. Thus, all rate constants calculated for the Blue LED experiment could not be corrected for adsorption.

**Figure 10:**
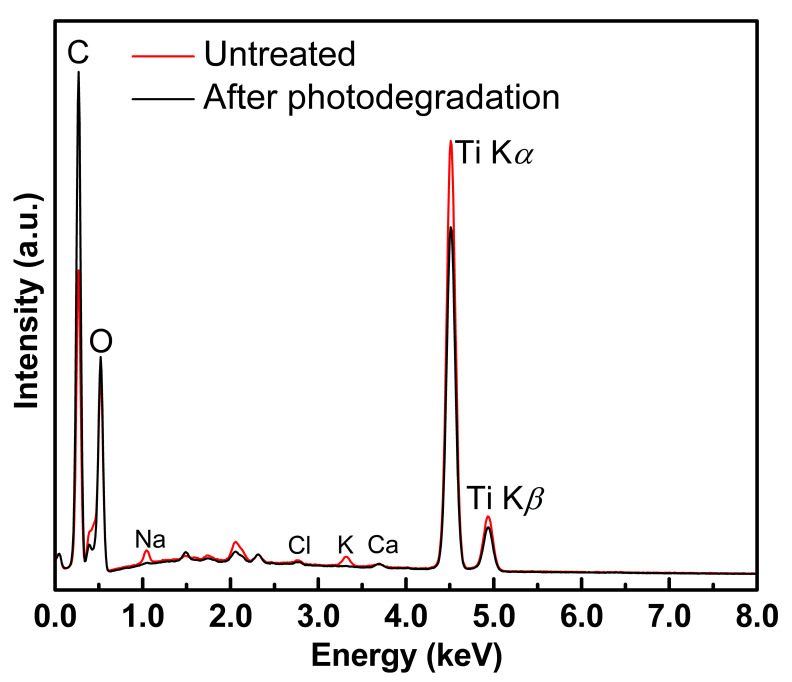
EDS spectra on the surface of Fe/TiO_2_–cMDF (Blue LED).

Since the slabs used in this experiment were immersed in aqueous MB medium for the entire experimental period, a concern was recognized that some of the effective Fe/TiO_2_ was removed from the surface of Fe/TiO_2_–cMDF during photodegradation. Thus, elemental distribution and elemental analysis by SEM–EDS were conducted on the surface of Fe/TiO_2_–cMDF to determine the Ti distribution and content. In Figure 10 and Table 3, the results of the elemental analysis through EDS indicated that Ti was removed, from 16.85% to 11.32%, during the series of photodegradation. Again, the presence of Fe was hardly detected in the EDS spectra.

**Figure 11:**
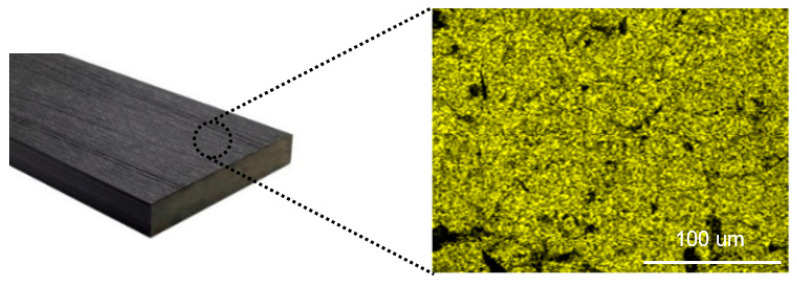
Elemental distribution images of Fe/TiO_2_–cMDF after photodegradation (Blue LED).

Although the Ti content was reduced on the surface of Fe/TiO_2_–cMDF, photodegradation of MB was still accomplished since effective Ti (Figure 11, Ti: yellow) remained dispersed on the surface of Fe/TiO_2_–cMDF after five cycles of photodegradation.

#### 3.4.2. Photodegradation Performance of Fe/TiO_2_–cMDF under Commercial LED Bulbs

**Figure 12:**
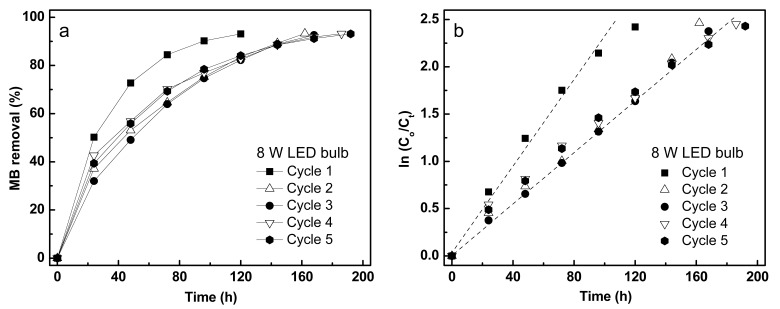
Photodegradation kinetics of MB under a commercial 8 W LED bulb: (**a**) MB removal and (**b**) normalized plot for pseudo-first-order.

**Figure 13:**
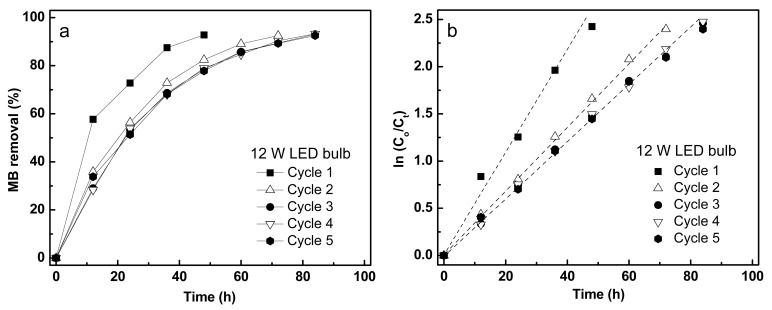
Photodegradation kinetics of MB under a commercial 12 W LED bulb: (**a**) MB removal and (**b**) normalized plot for pseudo-first-order.

After confirming the photocatalytic ability of Fe/TiO_2_–cMDF under Blue LED, photodegradation of MB was tested under two different commercial LED bulbs (8 W, 12 W) for five cycles. A photodegradation cycle was considered to be completed upon reaching 93% removal of MB. The kinetics results of MB removal under commercial LED bulbs are shown in Figure 12; Figure 13, and Table 4. Both photodegradation experiments followed pseudo-first-order kinetics (Figure 12b and Figure 13b), surprisingly different from the results of the photodegradation under Blue LED. The rate constants for the Blue LED experiment cannot be compared to the rate constants for the photodegradation experiments using commercial LED bulbs as pseudo-first-order (exponential) kinetics and pseudo-second-order (hyperbolic) kinetics are different functions.

Similar to the Blue LED experiment, the photodegradation under commercial LED bulbs was also direct photodegradation without pre-adsorption. Thus, in order to calculate the actual photodegradation rate constants, the effects of adsorption must be considered.

For the 8 W LED, the rate constants for the actual photodegradation after correction of the adsorption rate constants showed similar rate constants (12.0 × 10^−3^/h) except for the second cycle (9.5 × 10^−3^/h). The fourth and fifth cycles for photodegradation were not quantitatively corrected because a fourth and a fifth cycle for adsorption were not performed. Nonetheless, the rate constants for the fourth and fifth photodegradation cycles were retained at 12.6 × 10^−3^/h and 12.4 × 10^−3^/h, respectively.

For the 12 W LED, the corrected rate constant for the first cycle was found to have the fastest photodegradation performance at 41.4 × 10^−3^/h. However, after the first cycle, the rate constants for the second cycle to the fifth cycle remained consistent at ~28.5 × 10^−3^/h. Table 5 shows the corrected rate constants for the photodegradation under commercial LED bulbs.

**Figure 14:**
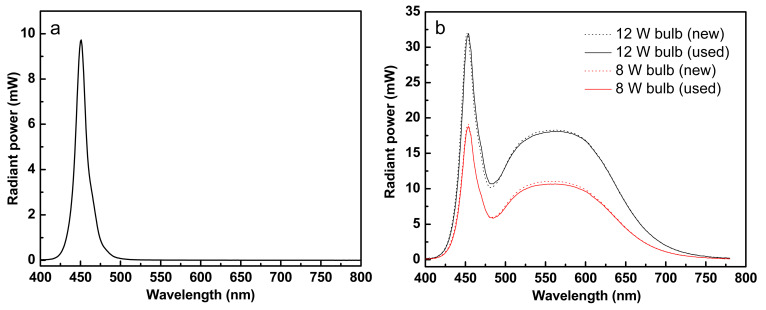
Radiant power of (**a**) Blue LED and (**b**) commercial LED bulbs.

The commercial 12 W LED bulb (41.4 × 10^−3^/h) exhibited a faster photodegradation performance than that of 8 W LED bulb (12.0 × 10^−3^/h). Comparing the rate constants of their first cycles, the 12 W LED bulb is 3.5× faster than an 8 W LED bulb. In fact, this was already expected since the radiant power of a 12 W LED is larger than 8 W LED, as shown in Figure 14b. Although the results of the elemental analysis by EDS showed a slight decrease in Ti content (Table 6), the photodegradation rate constants after the first cycle were almost the same. Therefore, it was considered that a small amount of Ti reduction during the experiment did not significantly affect the photodegradation rate constants, in contrast to the Blue LED experiment.

### 3.5. Characteristics of Blue LED and Commercial LED Bulbs

Blue LED emits a narrow wavelength at 450 nm, which corresponds to energy of 2.76 eV (Figure 14a). Commercial LED bulbs emit a narrow wavelength at 450 nm and a broad wavelength at 570 nm, the latter being the dominant wavelength, corresponding to energies from 2.18 to 2.76 eV. The energy supplied by Blue LED or commercial LEDs was sufficient for the bandgap energy requirement of Fe/TiO_2_–cMDF, which is 2.60 eV as calculated from the Kubelka–Munk function shown in Figure 7b.

As expected from the results shown in Figure 14b, the radiant power of a 12 W LED bulb is higher than an 8 W LED bulb (solid lines). In addition, the plot compares the radiant power of a used LED bulb (solid lines) to a new LED bulb (dotted lines). For an 8 W LED bulb, the radiant power decreased by 3.31% at 570 nm while a 12 W LED bulb showed only a 1.1% decrease at the same wavelength. The reason for this outcome is that the 8 W LED bulb was on for 834 h, but the 12 W LED bulb was on for only 388 h. These results suggest that commercial LED bulbs with higher wattage are efficient light sources for the continuous treatment of dye wastewater using Fe/TiO_2_–cMDF.

### 3.6. TiO_2_-Treated and Metal-Doped TiO_2_-Treated Wood and Wood-Based Composites

Table 7 shows related literatures on TiO_2_-based wood and wood-based composites. These composites remove pollutants through the combined action of adsorption and photodegradation. Several TiO_2_ wood and wood-based composites [6,18,19] removed VOCs while [14,15,20,21,22] removed organic dyes. Adding to this list are the metal-doped TiO_2_ wood and wood-based composites [13,23,24] which removed VOCs while [25,26] removed organic dyes. The results of the authors’ previous work [13] may not be comparable to this work since the TiO_2_-cMDF was subjected to a full cycle of adsorption prior to photodegradation and the radiant power of the light sources was different. In addition, the mechanism of photodegradation for anatase-type TiO_2_-cMDF/UV light and rutile-type Fe/TiO_2_-cMDF/visible light is distinct from each other [8,27]. Although not comparable, Fe/TiO_2_-cMDF is a more economically feasible material for industrial applications due to its utilization of visible light.

## 4. Conclusions

The photodegradation of MB by Fe/TiO_2_–cMDF was performed under Blue LED and commercial LED (8 W, 12 W) bulbs. The adsorption and photodegradation experiments were conducted separately. The photodegradation experiments were performed without pre-adsorption using a fresh set of Fe/TiO_2_–cMDF slabs under a dark condition. The photodegradation rate constants were corrected by subtracting the adsorption rate constants, except for the Blue LED experiment due to its difference in kinetics. Photodegradation under Blue LED followed pseudo-second-order kinetics. The fast rate constant for the first cycle was presumably caused by simultaneous adsorption and photodegradation. After the first cycle, the rate constants were very similar. Photodegradation under commercial LED bulbs followed pseudo-first-order kinetics. For 8 W LED bulbs, the corrected rate constants for the actual photodegradation showed similar rate constants. For 12 W LED bulbs, the corrected rate constant for the first cycle was found to have the fastest photodegradation performance. The rate constants for the second to fifth cycle remained constant. The energy supplied by Blue LED or commercial LED was sufficient for the bandgap energy requirement of Fe/TiO_2_–cMDF. To improve the photocatalytic performance of Fe/TiO_2_–cMDF for the continuous treatment of dye wastewater, the number of slabs must be increased.

## Figures and Tables

**Table 1:** Adsorption kinetics of MB removal (daylight and dark conditions).

		Daylight	Dark	Difference
	Cycle	*k*_1_ (×10^−3^/h)	*k*_1_ (×10^−3^/h)	∆ (×10^−3^/h)
Adsorption	1	12.9	8.5	4.4
	2	6.7	4.4	2.3
	3	3.0	2.6	0.4

**Table 2:** Photodegradation kinetics of MB removal under Blue LED.

	Cycle	*k*_2_ (×10^−3^/ppm∙h) *	t_99_ (h)	t_99_ (d)
Photodegradation	1	10.7	96	4.0
	2	3.9	270	11.25
	3	2.6	414	17.25
	4	2.6	480	20.0
	5	2.1	672	28.0

* Pseudo-second-order rate constants (uncorrected).

**Table 3:** Elemental analysis on the surface of Fe/TiO_2_–cMDF (Blue LED).

Element	Atomic Percentage	
	Untreated	After Photodegradation
C	42.97 ± 3.74	54.22 ± 4.95
O	39.46 ± 2.45	34.30 ± 2.93
Ti	16.85 ± 1.01	11.32 ± 3.03
Fe	0.02 ± 0.01	0.01 ± 0.01

**Table 4:** Photodegradation kinetics of MB removal under commercial LED bulbs.

		8 W LED		12 W LED	
	Cycle	*k*_1_ (× 10^−3^/h)	t_99_, h (days)	*k*_1_ (× 10^−3^/h)	t_99_, h (days)
Photodegradation	1	20.5	120 (5.0)	49.9	48 (2.0)
	2	13.9	162 (6.75)	33.7	72 (3.0)
	3	13.9	174 (7.25)	29.5	84 (3.5)
	4	12.6	186 (7.75)	29.9	84 (3.5)
	5	12.4	192 (8.0)	28.0	96 (4.0)

**Table 5:** Corrected photodegradation kinetics of MB removal under commercial LED bulbs.

		*k* (× 10^−3^/h) for 8 W		*k* (× 10^−3^/h) for 12 W		
Cycle	*k*_1_	*k*_1_*,* Dark	*k*_1_, Corrected	*k*_1_	*k*_1_*,* Dark	*k*_1_, Corrected
1	20.5	8.5	12.0	49.9	8.5	41.4
2	13.9	4.4	9.5	33.7	4.4	29.3
3	13.9	2.7	11.2	29.5	2.7	26.8
4	12.6	-	12.6	29.9	-	29.9
5	12.4	-	12.4	28.0	-	28.0

**Table 6:** Elemental analysis on the surface of Fe/TiO_2_–cMDF (commercial LED bulbs, 12 W).

Element	Atomic Percentage	
	Untreated	After Photodegradation
C	42.97 ± 3.74	53.12 ± 7.04
O	39.46 ± 2.45	32.61 ± 4.27
Ti	16.85 ± 1.01	14.06 ± 3.11
Fe	0.02 ± 0.01	0.02 ± 0.01

**Table 7:** TiO_2_-based wood and wood-based composites.

Composites	Removal	Efficiency	Light Source	Reference
TiO_2_-cMDF	Formaldehyde (g ^a^)	99%, 24 h	UV-A lamp	[6]
	Toluene (g)	99%, 5 h	UV-A lamp	[6]
	MB (aq ^b^)	99%, 348 h ^c^	UV-C lamp UV-A LED	[14,15]
TiO_2_-woody composite	Formaldehyde (g)	99%, 3 h	UV lamp	[18]
TiO_2_-wood template	Formaldehyde (g)	20%, 5 h	UV lamp	[19]
TiO_2_-wood template	Rhodamine B (aq)	90%, 3 h	UV lamp	[20]
TiO_2_-MDF biochar	MB (aq)	86%, 3 h	UV lamp	[21]
TiO_2_-bleached wood	MB (aq)	99%, 7 h	UV sunlight	[22]
				
Fe/TiO_2_-wood	Formaldehyde (g)	93%, 5 h	Visible lamp	[23]
Ag/TiO_2_-wood substrate	Formaldehyde (g)	92%, 10 h	Visible LED	[24]
Co/TiO_2_-MDF	MB (aq)	80%, <1 h	UV-A lamp White/Green lamp	[25]
WO_3_/TiO_2_ -wood fiber	MB (aq) Rhodamine B (aq)	97%, <1 h	UV-A lamp	[26]
Fe/TiO_2_-cMDF	Toluene (g)	99%, 15 h	Fluorescent lamp	[13]
	MB (aq)	93%, 48 h	Blue LED LED bulbs	This work

^a^ g: gas, ^b^ aq: aqueous, ^c^ The composite was subjected to a full cycle of adsorption prior to photodegradation.

## Data Availability

Most of the data used during the preparation of the manuscript are included in the Results and Discussion section.
